# Supplementary material for: Negative Perception of Bats, Exacerbated by the SARS-CoV-2 Pandemic, May Hinder Bat Conservation in Northern Uganda
Source: Sustainability. Author manuscript; Available in PMC 2025 Aug 13. (PMC12345411; doi:10.3390/su142416924)
Supplement: 1 [file NIHMS2047932-supplement-1.pdf]

Negative perception of bats, now exacerbated by the SARS-CoV-2 pandemic, may hinder bat conservation in Northern Uganda

Imran Ejotre [1,2,*], DeeAnn M Reeder [3], Kai Matuschewski [1], Robert Kityo [4] and Juliane Schaer [1,*]

Supplementary Materials

Text S1. Questionnaire - Knowledge and awareness about bats in Northern Uganda

Table S1: Overview responses of individual and group interviews. 2A,B

Table S2: Interviews elderly

Text S2: Extended results – sensitization campaign

Supplementary Materials 1: Questionnaire - Knowledge and awareness about bats in Northern Uganda

KNOWLEDGE AND AWARENESS ABOUT BATS IN NORTHERN UGANDA

The purpose of this questionnaire is to collect data on the knowledge and awareness about bats in northern Uganda as part of a proposed initiative aimed at protecting bats and their roosts in Uganda. You may answer the questions to the best of your knowledge.

General information

Date: ______ Community: ____________
Gender: **M/F** Age: [>18years __; 18-30years__; 31-40years__; 41-50years__; >50yeays___]
Occupation: _____________

Information on bats

1. Do you have bats in your area? **YES/NO**

2. How many different types of bats have you seen or do you know in the area?
   ....
   a. Can you describe each type using color, size, facial structure, what they eat, etc.?
      ....
      ....

3. Bats are birds. **TRUE/FALSE**
4. If true or false, what similarities or differences (depending on your answer) do you know between the two?
   ………………………………………………………………………………………………………
   …………………………………………………………………………

5. What kind of places in your area can bats and bat roosts be found?
   ………………………………………………………………………………………………………
   ………………………………………………………………………….

6. Do bats benefit humans? **YES/NO**
   a. If yes, please list/explain the uses/benefits of bats in your community:
      …..
      ....

7. Should bats be conserved? **YES/NO**
   a. If yes, what actions should be taken in your community in order to conserve bats?
      ....
      ....

   b. If no, why should bats not be conserved?
      ....
      ....

8. What stories does your community have about bats?

………
……………………………………………………………………

THANK YOU

Additional questions

9. How have people in your community related with or treated bats in the past?
………
………
……………………………………………………………………

10. Has this relationship changed over the years? **YES/NO**
    a. If yes, when did this change in attitude or relationship start and what has triggered this change?
………
………
……………………………………………………………………

11. What is your knowledge about the relationship of the diseases COVID-19 and Ebola with wildlife?
………
………
……………………………………………………………………

12. Where do you get your information about bats? (relatives/family, community leader, in the city, media (smartphone, internet, twitter, news, etc.)?
………
………
……………………………………………………………………

13. From our interactions today, have you learnt something about bats that you hadn't known before? What is it that you hadn't known about bats before?
………
………
……………………………………………………………………

14. How do you intend to relate/deal with bats in the future?
………
………
……………………………………………………………………

Table S1: Overview responses of individual and group interviews.

Table S1. (A) Respondents count (n) and frequency (%) of the responses for questions regarding basic knowledge about bats (Answers YES/NO). (**B)** Respondents count (n) and frequency (%) of the responses for questions regarding basic knowledge about bats (open questions).

**(A)**						
**Question** (number in the questionnaire)	**Individual interviews** (Participants, n=60)			**Focus groups (n=6)** (A=University academic staff; B= University students; C=Secondary School students; D=The elderly; E=Medical personnel; F=Rural community) (Participants, n=91)		
	**YES, n (%)**	**NO, n (%)**	**Do not know, n (%)**	**YES[1]**	**NO[1]**	**Do not know[1]**
Do you have bats in your area? (#1)	58 (97)	2 (3)	0 (0)	6/6	0/6	0/6
Do bats benefit humans? (#6)	34 (57)	26 (43)	0 (0)	6/6	3/6 (B,C,F)	0/6
Should bats be conserved? (#7)	28 (47)	27 (45)	5 (8)	6/6	6/6	0/6
	**TRUE, n (%)**	**FALSE, n (%)**	**Do not know, n (%)**	**TRUE[1]**	**FALSE[1]**	**Do not know[1]**
Bats are birds. (#3)	7 (12)	52 (87)	1 (1)	0/0	6/6	0/0

**(B)**		
**Question** (number in the questionnaire)	**Individual interviews** (Participants, n=60) Answers highest to lowest n (%)	**Focus groups (n=6)** (A=University academic staff; B= University students; C=Secondary School students; D=The elderly; E=Medical personnel; F=Rural community) (Participants, n=91)
How many different types of bats have you seen or do you know in the area? (#2)	Two: 34 (57); one: 11 (18); three: 7 (12); no idea: 5 (8); four: 3 (5)	Three (A,E); four (D,F); two (B); one (C)
Can you describe each type using color, size, facial structure, what they eat, etc.? (#2A)	By size: small: 23 (38); large: 22 (37); medium: 5 (8); do not know: 10 (17)	By size: big/small (A,B); do not know (C)
	By color: (dark) brown: 39; black/dark: 23; grey: 10; orange/yellow winged: 5	By color: brown/black (A,B); do not know (C)
	Facial structure: long/dog-like: 3; small/short face: 3	Facial structure: do not know (C)
	By food eaten: fruits: 15; insects/flies/worms: 14	By food eaten: do not know (C)
	other: -	other: House & cave bats, bats that live in pit latrines, big dog looking bats live in big groups on trees and medium size dog looking bats live in trees (E,F)
Bats are birds. If **TRUE** or **FALSE**, what similarities or	produce young alive/lactating vs lay eggs: 44; teeth vs beak: 17; no idea: 7; nocturnal vs diurnal:	produce young alive/lactating vs lay eggs (A-F); teeth vs beak (A,B,D); fur vs feather (A,B); hung upside down vs upright sitting (A);

differences do you know between the two? (#4)	3; fur vs feather: 3; hung upside down vs upright sitting: 1	wings are webs that connect fingers to feet (F,F)
What kind of places in your area can bats and bat roosts be found? (#5)	big trees in homes/community: 52; house roofs: 48; caves: 17; pit latrines: 12; banana/coconut leaves: 6; shrubs and bushes: 5; no idea: 3; wild forests: 1	House roof ceilings, tree branches (A-F); caves (A,B,D,E,F); pit latrines (A,B,C,D,F); banana/coconut leaves (A); shrubs (D); crevices (A)
Do bats benefit humans? If **YES**, please list/explain the uses/benefits of bats in your community. (#6A)	no idea: 22; feed on insects that carry diseases/destroy crops: 16; bats are a delicacy to some people: 14; seed dispersal/improve plant genetic diversity: 14; roles in nature not known to humans: 8; aesthetic roles/tourist attraction: 5; medicinal (backache) and for intelligence: 3; bat guano as organic manure: 3; provide signal to fruiting seasons of tree species: 2; signal which tree fruits could be edible by humans**: 2; indicators of environmental health: 2	Fruit/Seed dispersal (A,B); pollination of crops/tree species (A); many ecosystem functions we do not know (A); no idea (C); Bats alert of fruiting seasons of different tree species when they drop fruits under their roosts; tell which wild fruit can be edible (if bats eat, probably not poisonous) (D,F); Every creature has its roles/benefits that should not necessarily be measured in relation to humans (E)
Should bats be conserved? If **YES**, what actions should be taken in your community to conserve bats? (#7A)	protect and conserve roosting sites and habitats: 23; community awareness and sensitization campaigns: 10; gazette & protect natural habitats to conserve wildlife***: 8; ban tree cutting/plant trees to create habitats for bats: 4; control fumigation of bat roost sites: 2; stop bat hunting especially by children: 1	Bats offer vital ecosystem services to us, need to protect natural environments where bats live and particularly their established roosting sites including those in lands under private ownership (A); Protect forests so that bats can go back to forests, relocate bat roosts away from human environment peacefully, to avoid bats following big trees in homesteads leave many big trees in the community so that when bats are chased from trees in homes, they can find alternatives, otherwise they return (B); Yes, because they are living things and they need to exist. Only chase away those living in houses and in trees in homes (C); We should not cut all big trees in the forest to attract bats to live with us. Because caves are rare, human structures offer ready and better roosting sites for bats so if we want bats away, create similar structures in fields away from homes (D); Protect natural habitats & roost sites located some distance from human habitation (E); Humans should not destroy every place so that bats can stay where they used to stay many years ago (F)
Should bats be conserved? If **NO**, why should bats not be conserved? (#7B)	transmit many diseases (e.g., Ebola, Covid-19): 20; Make places dirty/smelly (poop): 13; destroy our fruits: 10; spread bedbugs/lice to homesteads close to roosts: 3; noisy roosts near a home/poop on people during fly-out: 3; bring bad luck/ill omen: 3; bats are ugly and scary: 3; do not see any good use in them: 3; bats are used in witchcraft: 1	smell in houses, destruction of fruits, spread of bedbugs and bat lice, denying shades due to poop on people, ugly and scary appearance of bats, association with witchcraft, diseases like Ebola, Covid-19 (B); no beneficial roles (F)

(A) [1]answer by some or all members of the whole focus group (categories A-F); (B) ** Fruits eaten by bats are probably safer for human consumption; *** for their sake and for future generation; Focus groups: A = University academic staff (*n* = 8: 2f, 6m); B = University students (*n* = 15: 6f, 9m); C = Secondary school students (*n* = 16: 8f, 8m); D = The elderly >50 years (*n* = 15: 6f, 9m); E = Medical personnel (*n* = 20: 12f, 8m); F = Rural community (*n* = 17: 4f, 13m); n = number, f = female, m = male

Table S2: Interviews elderly. Participants ≥ 50 years that accepted to answer additional questions (n=16/22).

**Question** (number in the questionnaire)	**Answer**
How have people in your community related with or treated bats in the past? (#9)	Little relation because big trees were so many in the bush and bats mostly stayed in the bush. In the past, homesteads were scattered, people were few and there were plenty of forested areas for bats to stay in. No connection with bats. They lived in the bush and people had no interaction with bats. Bats not common around homes. Bats in houses would be chased away. Bats were not present in communities, only seen rarely.
Has this relationship changed over the years? **YES/NO (#10);**<br>If **YES**, when did this change in attitude or relationship start and what has triggered this change? (#10A)	YES (n = 12); NO (n = 4);<br>Less than 10 years ago (n = 0), 10 – 20 years ago (n = 11), Over 20 years ago (n = 1)
What is your knowledge about the relationship of the diseases COVID-19 and Ebola with wildlife? (#11)	Both Covid-19 and Ebola are said to be spread by bats. Diseases like COVID 19 and Ebola have been attributed to bats. Not specific but some wildlife is said to transmit Covid-19 and Ebola. Bats are mechanical vectors to rabies; their urine is very corrosive and destroys wood and metal. The guano is filthy. Ebola is harbored by wild animals like baboons and bats, while COVID-19 is harbored by domestic animals and humans. Many people don't know about the link between diseases and bats except town dwellers. I don't know much about bats. I have no knowledge about it.
Where do you get your information about bats? (relatives/family, community leader, in the city, media (smartphone, internet, twitter, news, etc.)? (#12)	Each respondent could name multiple sources:<br>Radio (n = 6), TV (n = 1), Internet/social media (n = 5), Relative/family member (n = 5), Community leader/member (n = 6), Public posters (n = 1)
From our interactions today, have you learnt something about bats that you hadn't known before? What is it that you hadn't known about bats before? (#13)	No direct link between bats and these diseases in humans. Pollination by bats, insect population check, seed dispersal. I didn't know that there are many types of bats, some weighing up to 1kg. Didn't know bats have different types. I did not know that bats can be useful to humans. Didn't know bats can be vectors to diseases. I didn't know that bats have teeth, I thought they were birds with wings. I didn't know there are many types and bats and the roles they play.
How do you intend to relate/deal with bats in the future? (#14)	*Negative comments:*<br>Bats should be eradicated from human environments because they spread bedbugs and bat lice to humans that bite and may cause disease. Bats and humans should not stay together. Solutions should be found to ensure that bats stay away from people from a public from public health perspective and agric perspective. I intend to keep bats out of my surroundings because as a farmer, I grow fruits like mangoes that bats destroy.<br>Environmental advocates should keep the bats where they are supposed to be by planting trees in the bush to attract them there. Otherwise, bats should be chased away via chemical sprays. Bats are treated as pests destroying fruits and the community still feels that they should be chased away or killed. I don't want to see bats in my area since they cause diseases. Avoid contact with bats to avoid spread of diseases. If I see a bat near my house, I will kill it straight away.<br><br>*Positive comments:*<br>Conserve the bats in caves and trees but destroy the ones in houses.<br>As creatures like us, bats should be conserved and promoted for tourism.<br>I want to keep bats and other animals.<br>We should friendly coexist with bats.

Note: There were 22 participants over 50 years of age and treated as 'Elderly'. However, those that responded to the additional questions specifically targeting this age group were 16.

Text S2: Extended results – sensitization campaign

Sensitization campaign and intervention of actions against bats

Specific actions against bats were planned in the communities in Koboko, Yumbe and Moyo districts of the study area around October 2020 as people were concerned about the links between bats and the SARS-COV-2 pandemic. People started to actively chase away bats from their homesteads and surrounding areas and marketplaces using items that cause loud shrill noises such as setting up local metal welding and carpentry stalls and burning plastics/motor vehicle tires under tree roosts to irritate the bats. Mainly colonies of fruit bats (*Epomophorus* spp., *Eidolon helvum*, Pteropodidae) were targeted with this approach to get rid of bats from shade trees close to homes or frequently used by humans. In Moyo district the COVID-19 taskforce led by the District Health Officer had planned to fumigate bats on the Mvule trees along Moyo Mission Road. The communities around the large roosting colonies of the fruit bat *Eidolon helvum* along Moyo Mission Road had plenty of negative experiences with the bats. They said apart from being noisy and making the area smell bad, the bats defecate everywhere on the nearby homes during emergence at dusk and their homes are continuously infested with bedbugs from the bats. Across much of the African continent, this species forms massive and loud aggregations with 100,000 up to 1,000,000 individuals in one colony [91], although the colonies within the study area were much smaller. At up to 350g, it is the second largest African fruit bat and is the only African bat species that migrates over long distances annually covering over 2,500 km from southern, eastern to western African countries [92-95]. A representative statement was given by a 60-year-old male from the Madi community in Moyo: "*Ultimately, people want to chase bats out of their vicinities. People along Mission Road (Moyo district) wanted to cut down the trees along this road that bats have used as roost for decades but have failed because Moyo Catholic Mission has always refused. Our hope now comes as this road is going to be upgraded into butamin (tarmac) and that the trees will finally be cut down. Meanwhile we have been burning motor tyres, shooting the bats with catapults, and making shrill noise to chase the bats away, which has mainly worked*." The communities in Moyo had no kind words for bats and hoped that the pandemic was the blessing they had waited for so long for the town council to finally heed to their complaints and cut down all the majestic Mvule trees (*Chlorophora exelsa*) along the Mission Road planted long ago by the Christian missionaries. Some members still hoped that should their cries fail to yield results now the trees will eventually go when that same road which leads to the South Sudan border will be upgraded to butamin (tarmac). The district entomology officer Moyo explained that bat urine is acidic which destroys peoples' house ceilings and branches of trees. He provided examples of trees in townships that served as bat colonies and whose branches had all dried out eventually leading to the death of the trees. He also showed examples of houses whose timber and ceilings broke down after being colonized by house bats for a long time. Coupled with the losses bats inflicted on agricultural produce like fruits, he vehemently advocated for the fumigation of all bat colonies be they in houses or trees. The rumors that bats are responsible for the outbreak of SARS-COV-2 pandemic perfectly drove his point home. He argued that it is not his responsibility to advocate for the protection of the bats. His colleagues in the environment department should see to it that the natural environment is restored, and bats go back to where they came from. This officer's influences aided the Moyo District COVID-19 Task Force team's push for a supplementary budget at the district local government council to fumigate bat roosting sites in human occupied areas especially within the town council. This officer and a few other respondents (particularly some university lecturers) were the group that either researched and read about bats or had some exposure to talks on bats that were able to mention some bat species by name, such as *Eidolon helvum*, molossid bats or knew about their diets (insect eating, fruit eating bats).

Similarly, the management of the Midigo hospital in Yumbe district (Midigo Health IV) had decided to either fumigate all bats in the mango trees (*Epomophorus* spp.) or cut down all trees on the hospital premises. The shades of the mango trees on the hospital premises are used by outpatients and inpatient attendants as resting places. Initially, when the COVID-19 pandemic guidelines of maintaining physical distance of 1.5 meters and wearing face masks came out, the outpatient lobby was not enough to accommodate the many patients at the hospital. Thus, the hospital management decided to use the numerous mango tree shades as clerking areas for outpatients, which met the social distance requirements. However, when the origin of the pandemic virus was associated with bats, people were scared about sitting directly below bat roosts and feared getting the virus via aerosol transmission. This led to the hospital management meeting that resolved to fumigate or cut down the roosting trees to reduce chances of contact with patients and hospital staff and address the fear of the patients.

The sensitization campaign was carried out in Koboko, Yumbe and Moyo districts in Northern Uganda. The leading author (IE) used printed copies of the booklet "Living Safely with Bats" and presented information about bat diversity, ecology, diseases and how to live safely with bats to members of the communities. The impression was that people were curious and the presentations were followed by several questions, e.g.: "How safe are we now as we have been staying close to these bats for some time?", "If bats are not confirmed to be the direct source of coronavirus as you have stated, why should we believe you and not the information we receive from radios and people?", "Can we temporarily chase the bats away as we prepare safer ways to share spaces with them?". Usually, the people were interested in the booklet, and copies were distributed. Several in-depth conversations took place with the communities, community leaders, and members of institutions whose premises have colonies of bats (Figure 5).

Generally, the sensitization campaigns were well received and attended (Figure 5). No incidences of hostility towards the sensitization team were registered, and the curiosity of the participants through questions were handled with the evidence available including giving them copies of the booklet "Living Safely with Bats" that was adapted to the local environment (Figure 5A). As a result of the sensitization campaign, the planned actions against bats at the Midigo hospital and in Moyo were prevented. The planned fumigation and cutting of trees at Midigo hospital was not carried out. Instead, tents were set up under the mango trees for outpatients and visitors to prevent direct contact of people with bat urine/feces as suggested during the sensitization campaign (Figure 5B). Generally, the roosts at the hospital premises remained intact and stable. People went about with their business without minding the presence of bats in the trees they use as shade. In Moyo the planned fumigation was temporarily halted. It was reported that a fierce debate ensued in the district council between the COVID-19 pandemic task force team led by the district health team and supported by other technical departments like agriculture on the one hand pushing for the approval of a supplementary budget to procure fumigants and/or permit destruction of bat roosts especially in the densely populated urban areas, and the members of the department of environment and natural resources on the other hand opposing the destructive and non-targeted approach to containing the pandemic. The environment team backed up their defense with evidence from our sensitization campaigns and won the debate. Council sitting did not approve the supplementary budget and resolved to not allow anyone fumigate or destroy bat roosts unless they produced more convincing new evidence that bats are responsible for the spread of the pandemic virus. Talks with the Moyo District Health Officer indicated that he has temporarily halted the action against bats.
